# An Essay on the Changes Which the Cervix and Lower Segment of the Uterus Undergo in the Second Half of Pregnancy

**Published:** 1843-07

**Authors:** 


### Art. XI.

*Ueber die Veränderungen des Scheidentheiles und des unteren Abschnittes der Gebärmutter in der zweiten Hälfte der Schwangerschaft.* Von Dr. Fr. H. G. Birnbaum.—*Bonn*, 1841. 8vo, pp. 94.

*An Essay on the Changes which the Cervix and Lower Segment of the Uterus undergo in the second half of Pregnancy.* By Dr. F. H. G. Birnbaum.—*Bonn*, 1841.

The alterations of the cervix uteri have been long, and with propriety, regarded as affording one of the most valuable signs of the existence of pregnancy. Their importance would, however, be greatly increased if they afforded a means of judging of the date of pregnancy as well as of its existence, and many German writers on obstetric subjects have, since the time of G. W. Stein, described these phenomena as occurring with most unvarying regularity, and as indicating exactly the period of utero-gestation. In England, on the contrary, most authorities speak of these changes as observing no constant order, and caution the inexperienced from attempting to diagnosticate the stage of pregnancy from the condition of the cervix uteri, since, as Smellie observes, "The neck of the womb will in some be felt as long in the eighth as in others at the sixth or seventh month." This discrepancy of opinion on a subject of considerable importance did not give rise to any fresh investigations, but some espoused one side of the question, others a different one, and not a few qualified their statements, so that it was not possible to deduce from them any definite conclusion. Stoltz, indeed, made it the theme of an essay in his "Considérations sur quelques points relatifs à l'art des accouchements," and Kilian briefly notices it in his *Geburtslehre.* The latter differs both from Stoltz and from Stein, and makes some very just remarks on the circumstances which have led others into error; but the grand conclusion to which his observations lead, is that the whole question stands in need of a thorough critical investigation.

To this task Dr. Birnbaum has applied himself, and the tract before us contains the results of his inquiries, which appear to have been conducted with great painstaking and conscientiousness. He kept records of the frequently repeated vaginal examination of fifty-four primiparæ, and compared with them the results obtained in the case of women who had already given birth to children. The changes in the cervix uteri of multiparæ appear to take place with far greater irregularity than in persons who have never given birth to children; and the fissures in the os uteri of the former are a further source of confusion to those who wish to draw any inference as to the stage of pregnancy from an examination per vaginam. But there are circumstances which may lead to mistake, even in the case of women who are pregnant for the first time. Dr. Birnbaum observes in § 3, that sometimes the vagina is attached to the cervix uteri so low down, as to leave only a small portion of the lip of the uterus, perhaps not more than a line in length, projecting into its cavity. This low insertion of the vagina in general exists only at the anterior lip of the uterus, but occasionally its attachment around the posterior part of the cervix uteri presents the same anomaly, in which case a person may readily fall into the error of supposing pregnancy to be very far advanced, when in reality it is not by any means near its termination. In cases of this sort the true condition is at once detected by pushing up the roof of the vagina with the finger, when the cervix uteri will be felt above it long, firm, and but little developed. Another source of error of even more frequent occurrence is furnished by the development of the base of the cervix uteri, which produces an apparent shortening, distinguishable at once from real obliteration of the neck of the womb by passing the finger within its cavity.

The degree of shortening of the cervix uteri indeed appears liable to modifications from so many causes, that it affords but little clue as to the stage of pregnancy. In muscular and athletic women the lower segment of the uterus becomes developed less readily, and the shortening of the cervix uteri takes place at a later period than in females of a more delicate frame, while the firm and unyielding abdominal parietes prevent the uterus from sinking down into the cavity of the pelvis until an advanced period of pregnancy, or even until the commencement of labour. In such persons too the base of the cervix becomes enlarged, and the difference between apparent and real shortening of the neck of the womb is often extremely remarkable. In women of a directly opposite temperament, those in whom there is great laxity of fibre, and who are the subjects of leucorrheal discharges, it likewise often happens that the cervix remains long, cylindrical, very dilatable, and with an open os uteri for many weeks. Often indeed it is scarcely at all shortened till labour actually sets in, when its dilatation occurs in an almost mechanical manner. There are also other causes of a more local nature which modify the changes of the lower segment or neck of the uterus, as "the excessive distention of the uterus, pendulous abdomen, too great or too slight inclination of the pelvis, contraction or malformation or excessive width of the brim of the pelvis ; unnatural presentations of the child, or any cause which keeps the head high above the brim of the pelvis." (Sect. 8, p. 40.)

We must refer our readers to the work itself for an account of the effects produced by each of the above-mentioned causes, while we glance at another point on which great diversity of opinion has existed, namely the degree of opening of the os uteri during pregnancy, and the mode in which this opening is effected. Roederer, the Steins, Montgomery, and Kilian speak of a patulous state of the external os uteri, as occurring at an early stage of pregnancy, while Osiander, Baudelocque, and Stoltz hold a directly opposite opinion. Dr. Birnbaum's observations on this subject are both interesting and important:

"Usually," he says, "we find at an early stage of pregnancy, soon after the lapse of the first half, but at a period so uncertain that it cannot be stated positively, a commencing separation of the lips of the uterus which had been in contact up to that time. First of all a small shallow funnel-shaped depression is formed, which just admits the point of the index-finger, as may be seen by examination with the speculum. This gradually opens higher and higher so as to form a canal, narrowing towards the top and slightly curved in its course. As the opening advances, the tip of the finger reaches the superior aperture of the canal, and is prevented from advancing further only by a thin, almost membranous ring, which contracts firmly on every attempt to pass the finger beyond it. By degrees the edges of this opening yield to the finger and the cavity of the cervix uteri is now found to be a canal of uniform dimensions, at the end of which the membranes of the ovum may be reached." (Sect. 17, pp. 61-2.)

To this rule, however, there are many exceptions, and the opening of the cervix uteri from within outwards, as described by Stoltz, is occasionally observed, while in other cases the external os uteri will at one time be found most dilated, at another the internal, without our being able to assign any reason for the variation. Stoltz laid it down as a rule, that in first pregnancies the neck of the womb becomes effaced from within outwards, and that in subsequent ones the process takes place from without inwards. Dr. Birnbaum's researches have led him to the conclusion that this statement is altogether erroneous ; that the obliteration of the cervix uteri from within outwards is always an exception to the usual course of things, and that it is by no means characteristic of first pregnancies. It may indeed be asserted that there exists no characteristic difference between the changes in the uterus of primiparæ and multiparæ with the exception of those which are produced by fissures of the os and cervix, and their cicatrices. The opening of the cervix from within outwards occurs as well in first pregnancies as in the case of women who have had children ; in both it is unusual, but it is rarer in the latter.

The alterations in the form of the os uteri are regarded rather as characteristic of pregnancy than as indicating its stage. To one of these changes, the alteration of the form of the orifice from transverse to circular, great importance has been attached by some writers. Dr. Birnbaum examined the os uteri with the speculum in thirty-six women who were pregnant for the first time. In twenty-three the os uteri was a transverse opening, which retained completely the character presented by the unimpregnated uterus in two instances. In nine it was circular, and in four it approached the round form, but was oval rather than circular. These facts satisfactorily prove that the circular form of the os uteri cannot be regarded as of much value as a sign of pregnancy. Montgomery noticed that the os uteri often *felt* circular, and Dr. Birnbaum says that he has often convinced himself by an examination with the speculum that the orifice of the womb often *feels* circular when its form is in reality transverse. The apparently circular form of the os uteri depends in a considerable degree on the mucus which fills its cavity, and which often differs in quantity, thus producing frequent changes in the form of the mouth of the womb.

The above are the chief points illustrated in this essay; we could have wished that the facts it contains had been better arranged and stated in a more lucid manner. The first sixteen pages are occupied with details of the vaginal examinations on which Dr. Birnbaum grounds his statements; but their results are not thrown into tables or made in any way available to the reader, so that except in so far as they show that the author has not dealt in mere assertions, they might as well have been omitted. Fearing that some of his conclusions may have escaped our notice, we give, in Dr. Birnbaum's own words, the summing-up of his inquiries:

"1. Of all the changes induced by pregnancy, the relaxation of the cervix uteri is the most variable and uncertain. Usually, however, it takes place from without inwards, or from the point to the base, very rarely in the opposite direction, or from the base to the apex.

"2. With the relaxation of the cervix the opening of its canal is intimately connected. This, too, generally takes place from without inwards, very seldom from within outwards; but often it alternates from the one to the other. The mode in which it occurs is not characteristic of first or subsequent pregnancies, but the opening of the cervix from within outwards, which is always a rare phenomenon, appears to be much more unusual in multiparæ than in women who are pregnant for the first time.

"3. The shortening of the cervix uteri is partly apparent, owing to growth and distention, partly real, in which case it results from the disappearance of its middle part. Real shortening of the cervix is most distinctly recognized by thinning of the lower segment of the uterus, and shortening and widening of the canal of the cervix.

"4. The final obliteration of the cervix uteri is produced either by its walls being drawn apart in the progress of development of the lower segment of the uterus, or by a kind of process of inversion by which the internal undilated os uteri is driven through the external. This inversion is possible, though rare during pregnancy, but is by no means unusual in labour if the lower orifice is very lax, and the upper very resistent.

"5. The internal and external os uteri then never pass into each other, but one orifice alone remains, which is in most cases the external os, but in some few instances the internal.

"6. With the exception of the fissures and cicatrices of the os uteri, there exists no invariable difference between the occurrences in first and in subsequent pregnancies. In the former, however, greater and more rapid changes usually occur in the lower segment of the uterus than are observed in persons who have already given birth to children." (pp. 83-4.)

